# Neural correlates of task and source switching: Similar or different?

**DOI:** 10.1016/j.biopsycho.2010.01.008

**Published:** 2010-03

**Authors:** Iroise Dumontheil, Sam J. Gilbert, Paul W. Burgess, Leun J. Otten

**Affiliations:** Institute of Cognitive Neuroscience, University College London (UCL), 17 Queen Square, London, WC1N 3AR, UK

**Keywords:** Event-related potentials, Executive functions, Cognitive control, Task switching, Internal/external

## Abstract

Controlling everyday behaviour relies on the ability to configure appropriate task sets and guide attention towards information relevant to the current context and goals. Here, we ask whether these two aspects of cognitive control have different neural bases. Electrical brain activity was recorded while sixteen adults performed two discrimination tasks. The tasks were performed on either a visual input (letter on the screen) or self-generated information (letter generated internally by continuing the alphabetical sequence). In different blocks, volunteers either switched between (i) the two tasks, (ii) the two sources of information, or (iii) tasks and source of information. Event-related potentials differed significantly between switch and no-switch trials from an early point in time, encompassing at least three distinct effects. Crucially, although these effects showed quantitative differences across switch types, no qualitative differences were observed. Thus, at least under the current circumstances, switching between different tasks and between perceptually derived or self-generated sources of information rely on similar neural correlates until at least 900 ms after the onset of a switch event.

## 1 Introduction

Cognitive control ensures that behaviour is adjusted in response to changing environmental demands. It relies on a collection of brain processes that support the guidance of thoughts and behaviours in accordance with internally generated goals or plans. One aspect of cognitive control that has been studied relatively extensively is the selection and implementation of “schemas” (Norman and Shallice, 1986) or “task sets” (Monsell, 2003). It is thought that any task involves a configuration of mental processing resources (Shallice et al., 1989). Appropriate task performance requires the recruitment of those resources that are appropriate for the particular context and environment. Driving, for example, relies on object recognition, spatial processing, manual control, and so on. The selection, implementation, and maintenance of task sets need to be controlled to prevent disruption of an ongoing task, without compromising the flexibility to rapidly execute other tasks when appropriate.

Processes associated with the reconfiguration of task sets have been studied with paradigms in which subjects switch between tasks. In a typical task switching experiment, subjects are trained on two tasks. Each requires attention to, and classification of, a different stimulus attribute. Subjects perform these tasks on different trials, thus necessitating them to switch tasks across trials. Various methods have been used to inform subjects of which task to perform on each trial (for a review, see Monsell, 2003). The critical comparison is between ‘switch’ trials (where the task on the current trial differs from that on the previous trial) and ‘stay’ trials (where the task remains the same). Questions about reconfiguration processes can be answered by observing how performance and neural activity differ across these two types of trial.

More recently, other aspects of cognitive control have become of interest (e.g. Wager et al., 2005, 2006). Burgess et al. (2005, 2007) proposed a distinction between perceptually derived and internally generated information in the context of the ‘gateway’ hypothesis to explain the function of rostral prefrontal cortex. Perceptually derived or ‘stimulus-oriented’ (SO) information refers to information taken in from the outside world (e.g. a concert or lecture). By contrast, self-generated or ‘stimulus-independent’ (SI) information refers to information “de-coupled” or not directly obtained from the environment, but generated internally (e.g. planning future actions, or metacognition such as thinking about one's thoughts or reflecting on past events). The gateway hypothesis proposes that rostral prefrontal cortex is involved in biasing the cognitive processing system towards one of these two types of information. That is, this part of cortex may regulate whether attention is directed towards SO or SI information, depending on which is most relevant for the current circumstances. This biasing sometimes occurs automatically, such as when attention is captured by the environment (e.g. a loud noise) or by self-generated information (e.g. an unresolved issue that keeps coming to mind). At other times, attention needs to be actively redirected to fulfil the current goals. Such a situation might arise when circumstances automatically attract attention in the wrong direction. For example, during a boring lecture when the mind tends to wander, attention needs to be actively directed towards the perceptually derived information coming from the speaker. Conversely, when a problem needs to be solved in a distracting environment, attention needs to be directed towards self-generated information.

Other theories of rostral prefrontal cortex also emphasize a role of this region in the processing of particular sources of information. Christoff and Gabrieli (2000) and Christoff et al. (2001, 2003) proposed that lateral rostral prefrontal cortex supports the manipulation of self-generated information, rather than, like the gateway hypothesis, limiting the role of this region to attending towards self-generated information. Independently, Pollmann et al. (2000, 2006), Pollmann (2001, 2004), and Weidner et al. (2002) used findings from functional magnetic resonance imaging (fMRI) studies of visual target search to argue that left lateral rostral prefrontal cortex supports the weighting of attention towards relevant target dimensions, in particular when such dimensions change from trial to trial. The latter proposal has some aspects in common with the gateway hypothesis, in the sense that it suggests that lateral rostral prefrontal cortex plays a role in allocating, or weighting, attention towards different types of information: perceptual dimensions for Pollmann et al., perceptually derived versus self-generated information in the gateway hypothesis framework.

The interest in the present experiment is in the distinction between perceptually derived and self-generated information as postulated by the gateway hypothesis (Burgess et al., 2005, 2007). We use the term “source switching” to refer to the process of biasing the cognitive system towards one of these two types of information. This definition does not specify a directional specificity for the switching process, because there is currently no evidence for differences between switching from perceptually derived to self-generated information and vice versa (Gilbert et al., 2005).

Source switching has several things in common with reconfiguring task sets (“task switching”). In both cases, sets of processes need to be coordinated to accommodate current and upcoming goals, in both cases switching can occur voluntarily or involuntarily (e.g. triggered by a distracting environment, mind wandering, or a dominant task set), and in both cases switching can help or hamper the task at hand. The interplay between source and task switching can be illustrated with the example of utilization behaviour in individuals with frontal brain damage. These individuals tend to perform actions associated with everyday objects, such as matches or scissors, even when these actions are contextually inappropriate (Lhermitte, 1983). This occurs because behaviour is dominated by inappropriate task sets that are automatically triggered by perceptually derived information. Thus, failures in both task configuration and source selection contribute to the inappropriate behaviour.

The apparent overlap between the processes associated with source and task switching begs the question whether similar, or different, neural processes underlie the two events. As mentioned earlier, task switching has been studied extensively with behavioural and neural measures (e.g. Arrington and Logan, 2004; Astle et al., 2008; Braver et al., 2003; Dove et al., 2000; Dreher et al., 2002; Mueller et al., 2007; Sohn et al., 2000; Wylie and Allport, 2000). Source switching, however, has only been investigated recently in relation to the function of rostral prefrontal cortex (Burgess et al., 2005, 2007; Gilbert et al., 2005). The aim of the current study was to directly compare the neural correlates of task and source switching.

Although source and task switching both involve cognitive control, they may make different demands on the brain. At first glance, source switching seems to entail the early allocation of attention to either perceptually derived information or self-generated information. Task switching primarily refers to a later level of processing, one that may orient attention towards particular properties of a representation (e.g. shape or colour) built on the basis of perceptually derived or self-generated information and that combines the central or perceptual information previously obtained with task rules and associated item–response mappings. Thus, source switching may affect the selection of information of either perceptual or self-generated origin, while task switching affects the choice of a response on the basis of that information. The neural correlates of these two types of switches may differ accordingly, either with respect to their timing, their nature, or both.

It should be noted that the distinction between task and source switching considered here differs from the distinction between intentional and attentional set switching proposed by Rushworth et al. (2002, 2005) or the similar distinction between rule switch and perceptual switch proposed by Ravizza and Carter (2008). What we refer to as task switching combines aspects of both attentional set switching (attending to a letter's curve or line) and intentional set switching (switching between two stimulus–response mappings). Source switching is thought to be domain and modality-independent, and thus not linked to changes between different properties of a stimulus. In contrast, the concept of attentional set switching (Rushworth et al., 2002, 2005) includes changes between different sensory modalities, different spatial locations and different perceptual characteristics.

Because of the interest in temporal as well as qualitative aspects of neural activity, we used event-related potentials (ERPs) to study the relationship between source and task switching. ERPs are small changes in the brain's electrical activity, time-locked to an event (see Handy, 2004, for an introduction). ERPs have high temporal resolution, making them particularly useful for studying transient processes such as those associated with task and source switching. In addition, although ERPs do not easily allow the specification of the brain regions associated with cognitive processes, they are able to establish differences in the qualitative nature of neural processes. This is accomplished by comparing the distributions of electrical activity across the scalp. In this way, ERPs can be used to discern whether task and source switching rely on similar, or distinct, sets of neural processes.

The usefulness of ERPs for studying cognitive control has been recognized previously (e.g. Astle et al., 2006; Barcelo et al., 2000; Rushworth et al., 2002, 2005; Wylie et al., 2008). There is a growing literature on ERPs and task switching. This literature is characterized by the diversity of paradigms used to address aspects of switching. Perhaps as a consequence, few consistent ERP effects have been observed when looking at ERPs elicited by the stimuli after switch cues, whereas switch cues have elicited more consistent effects. In general, ERP findings seem to be affected by the particular demands of the task (Jackson et al., 2001, 2004), the type of responses (Astle et al., 2008; Mueller et al., 2007), the interval between response and stimulus (Karayanidis et al., 2003), the distribution of switch trials (Wylie et al., 2003), and the use of cues (Swainson et al., 2006) or choice of task (Forstmann et al., 2007). Across studies, ERP correlates of task switching in response to cues or stimulus events usually take the form of slow, broad deflections that span a number of components rather than being focused on a single component. In paradigms that use pre-stimulus cues to signal task switches, switch cues relatively consistently elicit larger positive deflections over centro-parietal scalp sites (for reviews see Goffaux et al., 2006, 2008; Swainson et al., 2006; Travers and West, 2008; Wylie et al., 2008). This deflection may be related to the P300 family of components (e.g. Spencer et al., 2001). Following item onset, switch trials have been shown to elicit positive-going modulations over posterior scalp sites at different times (Jackson et al., 2001; Karayanidis et al., 2003; Mueller et al., 2007; Rushworth et al., 2005; Swainson et al., 2003, 2006). However, switch trials can also give rise to modulations over frontal scalp sites, which can be negative- or positive-going relative to stay trials following item onset (Jackson et al., 2001; Rushworth et al., 2002; Swainson et al., 2003). The inconsistencies across studies strongly suggest that there is not a single set of neural and cognitive processes that underlies task switching under all circumstances. It is unclear what other firm conclusions can be drawn at this point. Source switching, at least as defined by Burgess et al. (2007, 2005) and Gilbert et al. (2005), has to our knowledge not yet been studied with ERPs. Thus, whilst at an information processing level there exists a strong a priori link between traditional task switching paradigms and ‘gateway’ (Burgess et al., 2005, 2007) source switching tasks, there is as yet no supporting evidence from electrophysiological methods. This is important since such methods are perhaps most suited to examining the short time spans of these switching phenomena.

Accordingly, in the present study we contrasted source and task switching in a paradigm adapted from Rogers and Monsell (1995) and Gilbert et al. (2005). Subjects performed two tasks with different item–response rules. Each involved a judgment about an uppercase letter. In the “line” task, the decision was whether the letter contained a vertical straight line. In the “curve” task, the decision was whether it contained a curve. Importantly, the line and curve tasks were not simply a reversal of one another and could therefore not be reduced to a single task. This necessitated the switching of some or all aspects of the task sets when performing one versus the other. Within each task, we used two attention conditions (cf. Gilbert et al., 2005). In the SO condition, the task had to be performed on the letter presented on the screen. In the SI condition, the task had to be performed on a self-generated letter, using the sequence of the alphabet.

Task and source switching were combined in a 2 × 2 factorial design. Factors were task switching (present or absent) and source switching (present or absent), leading to four conditions (no-switching, source switching, task switching and double switching). The inclusion of a double switching condition allowed an assessment of any interaction effects. The conditions were varied across experimental blocks. Within a switching block, every other trial was a switch trial, following the alternating runs procedure (Rogers and Monsell, 1995; Kray and Lindenberger, 2000; Koch, 2005). The predictability of such a sequence limits the reliance on external cues, which is important to maximise the chances that subjects will focus on internally generated thoughts when they are supposed to. Stimulus presentation was self-paced with a response–stimulus interval of zero. This approach departs from typical ERP designs as it conflates activity associated with the current stimulus with that associated with the previous response. However, a self-paced sequence is crucial to equate the timings and cognitive processes across SO and SI conditions. When subjects go through the alphabet in their head, it is not possible to make them wait for a fixed duration between each letter/response. Furthermore, the absence of switch cues and the self-paced design may lead to additional prefrontal cortex recruitment. Indeed, prefrontal patients may demonstrate preserved performance in tasks where switches are cued, but impaired performance when the switches have to be self-generated (Burgess, 2000; Shallice and Burgess, 1991). To ensure we could make meaningful inferences despite the use of a zero response–stimulus interval, the relevant ERPs were equated with respect to the contribution of the response to the previous trial (see Section 2 for details). The main question of interest was whether the ERPs elicited by source and task switches differed in their timing, scalp distribution, or both. The analyses focused on the ERP responses within the first second of stimulus presentation to establish differences in early processes leading up to a successful switch.  

## 2 Methods

### 2.1 Subjects

Sixteen healthy volunteers (mean age 21 years, range 18–31, 11 women) took part in the experiment. All reported to be healthy with no neurological and psychiatric histories, and to have normal or corrected-to-normal vision. All but one subject reported to be right-handed. Four additional participants were tested but not included in the analyses. Two performed poorly during training (see exclusion criterion below) and were not tested with EEG. The other two were excluded from the analyses because of eye movement artefacts that could not be corrected. The experimental procedures were approved by the University College London and University College London Hospitals joint ethics committee. All subjects provided written informed consent prior to participation.

### 2.2 Stimuli and tasks

**Fig. 1:**
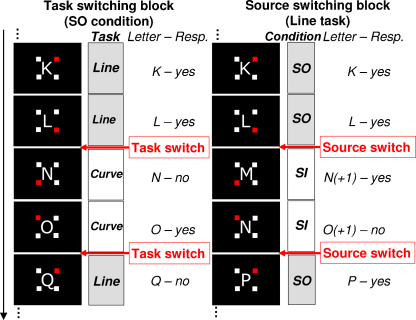
Examples of experimental sequences of trials. A single letter was presented on each trial, surrounded by four squares (the relative size of the letter compared to the screen is increased for display purposes). Three of the squares were white and the fourth red. The position of the red square changed across trials, rotating clockwise, and indicated the position of the trial in the sequence of alternating runs of two trials. Left: Example of a task switching sequence in a stimulus-oriented (SO) condition. Subjects had to either judge whether the letter on the screen contained a vertical line (the ‘line’ task) or whether the letter contained a rounded edge (the ‘curve’ task). The tasks switched every two trials. Right: Example of a source switching sequence with the line task. Subjects had to perform the task on either the letter presented on the screen (the SO condition) or on a letter generated internally by advancing the alphabetical sequence in their minds (the stimulus-independent or SI condition). Subjects switched between SO and SI conditions every two trials. (For interpretation of the references to color in this figure legend, the reader is referred to the web version of the article.)

Subjects saw series of letters presented in the centre of a computer monitor in a white uppercase Verdana font against a black background. Each trial involved the presentation of a single letter, surrounded by a small square at each of its four corners (Fig. 1). Stimuli were approximately 2° in height and width. On different trials, subjects performed one of two classification tasks on either the letter presented on the screen or a self-generated letter. In the ‘line’ task, subjects had to judge whether a letter contained a vertical line (e.g. K). In the ‘curve’ task, the decision was to judge whether a letter contained a rounded edge (e.g. S). Importantly, the line and curve tasks were not simply a reversal of each other, that is, the presence of a vertical line did not automatically inform about the presence of a curve and vice versa. The tasks could therefore not be performed with the same stimulus–response mappings. For both tasks, one of two response buttons had to be pressed with the left and right index fingers (responding hand balanced across subjects) as fast as possible without sacrificing accuracy.

Following Gilbert et al. (2005), the line and curve tasks were performed on either the perceptually derived information, i.e. the visual input (the stimulus-oriented, or SO, condition) or self-generated information (the stimulus-independent, or SI, condition). In the SO condition, subjects were asked to use the letter shown on the screen to make their decision. In the SI condition, they were to ignore the letter on the screen and instead generate a letter internally by continuing the alphabetical sequence in their head. In this condition, the line or curve judgment had to be made on the letter one step ahead (following the alphabet) of the letter presented on the screen. Importantly, because the sequence of letters was predictable when starting a pair of SI trials, perceptually derived information was not needed to discern this letter and make the correct decision.

Several measures were taken to encourage subjects to adhere to the requirement to attend to perceptually derived information in the SO condition and self-generated information in the SI condition. First, a predictable trial sequence was used. Every other trial was a switch trial, following the alternating runs procedure (Rogers and Monsell, 1995). This method was chosen to limit the reliance on external cues during SI switches. When the sequence of stay and switch trials is known, subjects can trigger the switching themselves and thus attend to self-generated thoughts (cf. Burgess, 2000; Shallice and Burgess, 1991). Alternating runs consisted of two trials only so as to maximise the number of switch events, and minimize the effect of stimulus repetition on ERPs. The small squares on the corners of the letters signalled where in the sequence of four trials (two consecutive pairs) subjects were, without directly cueing for a switch or stay event. This was accomplished by presenting one of the squares in a red colour on each trial in all conditions. The position of the red square changed across trials, rotating clockwise, and indicated the position of the trial in the sequence of alternating runs of two trials. The starting position was counterbalanced across subjects to start on the top left or top right corner.

Second, stimuli were presented in rapid succession to minimize the opportunity to think about processes not related to the task at hand. As soon as a response was given, the next letter was presented. Thus, the task was self-paced with a response–stimulus interval of zero. In the SI condition, subjects did not strictly have to wait for the next external stimulus given that the decision was based on a letter that was self-generated and therefore known in advance. The zero response–stimulus interval allowed an approximately equally rapid decision on SO trials. This approach departs from typical ERP designs, where stimulus and response are separated by an unfilled period. The ERPs elicited in the present study are a combination of neural activity associated with processing the letter on the current trial and activity associated with responding to the letter on the previous trial. This means that the wave shapes cannot easily be matched with known ERP deflections. To ensure meaningful inferences, the ERPs were equated with respect to the contribution of the response to the previous trial. For each type of switch (task switch, source switch, double switch) and for the no-switch condition, there was an equal proportion of SO and SI, and line and curve, conditions in the trial preceding the switch as well as the switch trial. The characteristics of the current and preceding trials were thus matched across conditions. Any differences across ERPs must therefore reflect differences in the way in which stimuli on the current trial were processed.

The line/curve tasks and SO/SI stimuli were combined into a 2 × 2 factorial design. Factors were task switching (changing between line and curve tasks; present or absent) and source switching (changing between perceptually derived and self-generated information; present or absent). The four resulting experimental conditions were performed in different trial blocks. Blocks could either be non-switching, in which case all trials in the block involved the same task and type of information (e.g. the curve task on SO stimuli). Or, blocks could be switching, in which case subjects alternated the task and/or type of information every two trials. In source switching blocks, subjects performed the same task on alternating stimulus types (e.g. the line task on SO versus SI stimuli). In task switching blocks, subjects alternated tasks on the same stimulus type (e.g. the line versus curve tasks on SO stimuli). Finally, in double switching blocks, both types of switching were combined. Accordingly, subjects alternated between line and curve tasks as well as SI and SO stimuli. Examples of trial sequences are given in Fig. 1.

The experimental sequences were constructed by assigning the letter ‘A’ to the first trial of the experiment and continuing the alphabetical sequence from then on. All letters were experienced in all the different conditions. To match the series across conditions further, the alphabetical sequence was sometimes advanced by two letters instead of one. This was necessary to prevent a decision being made on the same letter twice in a row. For example, a continuous alphabetical sequence of ‘K-L-M-N’ for SI-SI-SO-SO trials would have meant a decision on the letters ‘L-M-M-N’. To avoid the problem of repeated letters, the visual series skipped one letter during SI to SO switches in attention and double switching blocks (e.g. ‘K-L-N-O’ in the example above). Such skips before SO trials were also introduced in all other conditions to keep the sequences as similar as possible (see Fig. 1). In SO single task blocks, a skip was introduced every other letter (e.g. ‘*A*-B-*D*-E-*G*-H’); in SO task switching blocks, a skip was introduced at each task switch, i.e. every other letter. Note that these skips were predictable and affected SO, but not SI, switch trials. This means that (i) participants were more likely to pay attention to the visual information on SO switch trials, but (ii) could remain focused on the self-generated information on SI switch trials.  

### 2.3 Procedure

Subjects were first trained on all experimental task blocks. Training on a block terminated when 20 successive switch trials had been responded to correctly, corresponding to a minimum of 40 trials per practice block. If a subject did not reach this criterion, they were excluded from the experiment. The duration of the practice sessions ranged from 25 to 40 min, depending on the speed and accuracy of a subject.

The experiment proper was divided into ten experimental task blocks. Four of these consisted of no-switch blocks, one of each combination of task and stimulus (curve task on SO stimuli, curve task on SI stimuli, line task on SO stimuli and line task on SI stimuli). The remaining six blocks involved switching. Two blocks contained source switches (one with the curve task and the other with the line task), two contained task switches (one with SO stimuli and the other with SI stimuli), and two contained double switches (both involving the line/curve tasks and SO/SI stimuli). The order of the 10 blocks was counterbalanced across subjects, and each block was preceded by relevant instructions. Subjects could start a block whenever they were ready by pressing a response key. No-switch blocks consisted of 144 trials, separated into three parts with short breaks in between. Switch blocks consisted of 288 trials, separated into six parts. More trials were presented in switch than no-switch blocks because of the need to separate stay and switch trials in the former. ERP testing lasted on average 50 min, including short breaks between sessions. The whole experimental session lasted approximately 2–3 h (depending on how fast a subject responded).  

### 2.4 EEG acquisition and analysis

Electroencephalographic (EEG) activity was recorded continuously from 31 scalp sites using silver/silver-chloride electrodes embedded in an elasticated cap (montage 10; www.easycap.de/easycap/e/electrodes/13_M10.htm). The montage included three midline sites (1, 8, 14), 13 sites over each hemisphere (17, 19, 29, 30, 31, 33, 44, 45, 46, 47, 48, 49, 50 on the left and 9, 11, 22, 24, 25, 26, 36, 37, 38, 39, 40, 41, 42 on the right), and left and right mastoid sites. Midfrontal sites 8 and 20 were used as online reference and ground respectively. Vertical and horizontal eye movements were recorded bipolarly from electrodes placed above and below the right eye and on the outer canthus of each eye. All signals were amplified and band-pass filtered between 0.03 and 35 Hz (3dB roll-off). Digitization rate (12-bit resolution) was 250 Hz.

EEG epochs extending from 100 ms before item onset until 924 ms thereafter were extracted from the continuous record. Waveforms were reconstructed to represent recordings referred to linked mastoids and aligned to the 100 ms pre-stimulus interval. ERP waveforms were created for each electrode site and condition, and digitally smoothed, cutting frequencies above 25 Hz. Blink artefacts were minimized by estimating and correcting their contribution to the ERP waveforms via a standard regression technique (Rugg et al., 1997). Trials on which horizontal or non-blink vertical movements occurred were excluded from the averaging process, as were trials containing EEG drifts (±50 μV) or saturation of the analog-to-digital converter. Trials with incorrect responses were also excluded from analysis.

The primary question of interest was how neural activity associated with source switching differed from that associated with task switching. To this end, ERPs elicited by switch trials were compared across source switching, task switching, and double switching blocks. ERPs were collapsed across SO/SI stimuli and line/curve tasks (differences between these were of no interest in the present experiment as the focus was on each switching process as a whole). Note that the alternating runs procedure combined with a short response–stimulus interval does not lend itself to a direct comparison between switch and stay trials within blocks, as these trial types cannot be equated with respect to the contribution of the preceding response (switch trials are always preceded by stay trials and vice versa). Comparisons between switch trials across blocks, however, can be interpreted meaningfully as each trial type was equally often preceded by a stay event of the SO/SI stimuli and line/curve tasks.

Waveforms were quantified by measuring mean amplitudes across selected latency regions (relative to the 100 ms pre-stimulus baseline). The choice of these regions could not be guided by prior research, as no consistent task-switching effects have been identified. Instead, regions important for task switching in the present experiment were defined by initially comparing ERPs elicited during no-switch blocks with those elicited by switch trials in task switch blocks. These latency regions then formed the basis of comparisons between switch trials to address whether switch type consistently affected the ERPs across subjects. First, ERPs elicited by task switches were contrasted with ERPs elicited by source switches. Then, any interaction effects were assessed by considering ERPs to double switches. Mean amplitude values were submitted to repeated measures analyses of variance (ANOVAs), incorporating the Greenhouse–Geisser corrections for violations of sphericity (Keselman and Rogan, 1980). All 29 available electrode sites were initially entered into the analyses to minimize type I errors that may result from arbitrary selection of sites.

## 3 Results

### 3.1 Task performance

**Fig. 2:**
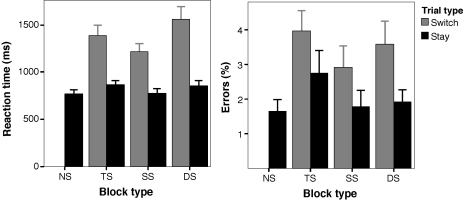
Task performance on stay and switch trials in each experimental block type, averaged across the line/curve tasks and SO/SI stimuli. Mean response time is shown on the left (ms, +SE), and accuracy is shown on the right (mean percent error, +SE). NS: no-switching, SS: source switching, TS: task switching, DS: double switching.

Fig. 2 illustrates the speed and accuracy with which the tasks were performed. Subjects generally performed well, with an average of over 95% accuracy. To confirm that participants used the information presented on the screen in SO trials, response times were compared between SI and SO trials in all conditions (no-switch, and switch and stay trials of the source switch, task switch and double switch blocks). Paired *t* tests demonstrated that participants were significantly slower in SI than SO trials in all seven conditions (all *t*(15) > 2.9, *p* ≤ .01).

Two types of task switching cost are usually observed (Monsell, 2003): (i) *mixing costs*, which correspond to a long-term cost of task switching, with poorer performance on stay trials of mixed task blocks compared to trials of single task blocks (Ruge et al., 2006); and (ii) *switch costs*, which corresponds to a poorer performance on switch trials than stay or repeat trials within mixed task blocks. To test whether such costs occurred in the present experiment, performance was compared across stay trials of single and mixed task blocks to assess mixing costs, and across stay and switch trials within blocks to assess switch costs. Trials were averaged across SO/SI stimuli and line/curve tasks, as directional changes were not of interest in the present experiment. Trials on which response times were more than three standard deviations from the mean were discarded from these analyses.

As expected, *switch costs* were observed in all switching blocks: switch trials were associated with more errors and longer response times than stay trials in task, source, and double switching blocks (paired *t* tests all *p* < .01). This indicates the presence of a significant transient cost of switching in all mixed task blocks. *Mixing costs* were analysed with repeated measures ANOVAs comparing trials of the no-switch blocks (single task blocks), and stay trials of the switching blocks (mixed task blocks). A significant effect of block type was observed for both accuracy (*F*(3, 45) = 4.6, *p* = .013, *ɛ* = .787) and reaction time (*F*(3, 45) = 5.0, *p* = .011, *ɛ* = .744). Pairwise comparisons indicated that mixing costs were observed in task switching (response time, *t*(15) = 2.4, *p *= .029; accuracy, *t*(15) = 2.8, *p *= .015) and double switching blocks (response time, *t*(15) = 2.3, *p *= .036), but not in source switching blocks (*p *> 0.6).

Possible differences in *switch costs* as a function of type of switching were evaluated with ANOVAs incorporating factors of trial type (switch/stay) and block type (source switching/task switching/double switching). Significant main effects of trial type for response accuracy (*F*(1, 15) = 19.3, *p* < .001) and reaction times (*F*(1, 15) = 35.5, *p* < .001) again confirmed the presence of switch costs. A main effect of block type in the ANOVA on response accuracy (*F*(2, 30) = 7.9, *p* = .003, *ɛ* = .887), combined with post-hoc analyses, indicated that subjects generally made more errors in task than source switching blocks. Importantly, trial type did not interact significantly with block type (*F*(2, 30) = 1.1, *p* > .3). For speed of responding, the main effect of block type was significant (*F*(2, 30) = 19.5, *p* < .001, *ɛ* = .925), as was the interaction between trial type and block type (*F*(2, 30) = 19.2, *p* < .001, *ɛ* = .811). Post-hoc comparisons showed that switch costs did not differ significantly between task (*M* = 521 ms, SD *=* 401 ms) and source switching (*M* = 442 ms, SD = 313 ms). However, both switch costs were smaller than the cost of double switching (*M =* 703 ms, SD = 434 ms, *p* < .001).

### 3.2 ERPs

#### 3.2.1 Task switching

**Fig. 3:**
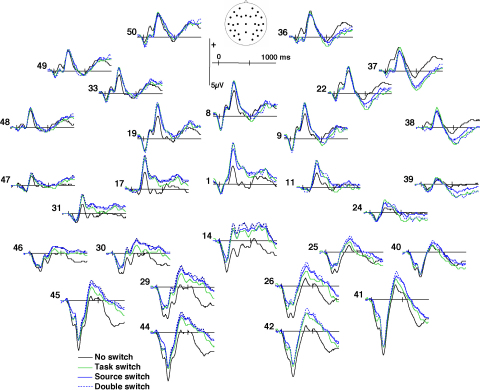
Group averaged ERP waveforms elicited by trials in no-switch blocks (black line) and switch trials in task switch (thin green line), source switch (thick blue line), and double switch (dotted blue line) blocks. Waveforms are shown for all 29 scalp sites; positive values are plotted upwards. (For interpretation of the references to color in this figure legend, the reader is referred to the web version of the article.)

**Fig. 4:**
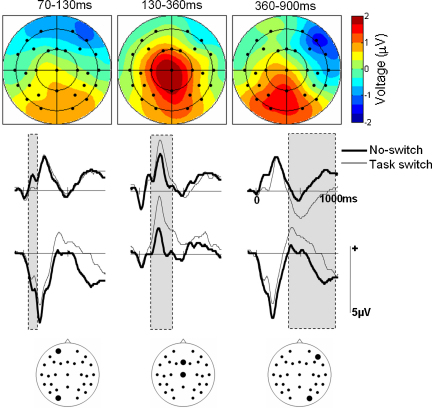
Effects related to task switching. Top: Scalp distributions of the observed ERP differences between no-switch and task switch trials (task switch – no-switch). Voltage spline maps showing the distribution of the effects across the scalp in the time intervals that formed the basis of the statistical analyses. The maps are scaled to the minimum and maximum across all differences to illustrate the distribution as well as size of the effects. Bottom: Group averaged ERP waveforms at representative electrode sites for no-switch (bold) and task switch trials. The positions of the sites are visualized on the electrode arrays. Grey shaded areas denote the latency intervals used for the statistical analyses.

Fig. 3 shows the group averaged ERPs elicited by switch trials in task, source, and double switching blocks along with the ERPs elicited in no-switch blocks. Differences between no-switch and switch trials are visible across the entire epoch from an early point in time. As explained in the Methods, the analyses initially focused on effects related to task switching. Fig. 4 illustrates these effects for a subset of electrodes. Considering time of occurrence and scalp distribution (see Handy, 2004, for an introduction on interpreting ERPs), differences related to task switching encompass at least three effects. At around 100 ms, switching between the line and curve tasks gave rise to waveforms that were more negative-going over frontal sites and more positive-going over occipital sites, relative to no-switch ERPs. Then, a positive-going modulation emerged over centroparietal sites until just before 400 ms. Task switching elicited a third effect during the remainder of the epoch, in the form of a more negative-going waveform over right frontal sites and a more positive-going waveform over posterior sites as compared with the waveforms elicited in no-switch blocks.

These effects were quantified by measuring mean amplitudes in the 70–130, 130–360, and 360–900 ms intervals. These intervals correspond with the times during which the above effects were most pronounced in the group average (a complementary analysis on consecutive 50 ms regions led to the same conclusions). Repeated measures ANOVAs incorporating factors of trial type (no switch versus task switch) and electrode site were used to evaluate whether differences in these intervals were consistent across subjects. The ANOVA on the 70–130 ms interval showed a significant interaction between trial type and electrode site (*F*(28, 420) = 7.3, *p* < .001, *ɛ* = .104). This interaction reflected the bipolar distribution of the early effect. Follow-up analyses on four representative frontal (36, 37, 49, 50) and posterior (41, 42, 44, 45) sites confirmed the reliability of both the negative-going frontal and positive-going posterior differences (main effects of trial type *F*(1, 15) = 12.1 and 8.7, respectively, both *p* < .01).

Significant interactions between trial type and electrode site were also found for the 130–360 and 360–900 ms regions (*F*(28, 420) = 5.3, *p* = .005, *ɛ* = .094, and *F*(2.3, 33.9) = 9.4, *p* < .001, *ɛ* = .081, respectively). The 130–360 ms region moreover demonstrated a main effect of trial type (*F*(1, 15) = 5.3, *p* = .036). The interactions reflected the central dominance of the positive modulation in the middle of the epoch and the bipolar distribution of the effect later on. In the latter case, follow-up analyses on four representative right frontal (22, 36, 37, 38) and posterior (26, 29, 44, 42) sites confirmed the reliability of both the negative-going frontal and positive-going posterior differences (main effects of trial type *F*(1, 15) = 17.6 and 8.0, respectively, both *p* < .013).

The scalp distributions of the three effects (see Fig. 4) were contrasted in an ANOVA incorporating factors of trial type, electrode site, and latency interval. This ANOVA revealed a significant three-way interaction (*F*(56, 840) = 5.2, *p* = .001, *ɛ* = .076), which remained significant after scaling the data with the max./min. method to remove overall amplitude differences across conditions and latency regions (McCarthy and Wood, 1985). Follow-up analyses showed that the distributions of all three effects differed from each other (all *p* < .05). Thus, in the present experiment, task switching was indeed associated with three distinct ERP effects.  

#### 3.2.2 Task versus source switching

Relative to the comparison between switch and no-switch trials, differences across the different types of switches were more subtle (see Fig. 3). At around 100 ms, switching between perceptually derived and self-generated information was associated with a less negative-going waveform over occipital electrodes than switching between line and curve tasks. Source switching also gave rise to a widespread positive-going modulation late in the epoch, with a maximum over posterior sites.

These effects were evaluated statistically in the same latency regions as defined for task switches in the previous section. This enabled a direct comparison across switch types (a complementary analysis on consecutive 50 ms regions again led to the same conclusions). For the 70–130 ms interval, an ANOVA incorporating factors of switch type (task versus source) and electrode site showed a significant interaction (*F*(28, 420) = 2.7, *p* = .036, *ɛ* = .150). Follow-up analyses on the four frontal (36, 37, 49, 50) and four posterior (41, 42, 44, 45) sites also used for task switch effects confirmed the visual impression that there was a difference between task and source switches at posterior (*F*(1, 15) = 7.7, *p* = .014), but not frontal (*F*(1, 15) < 1), electrodes. No significant effects emerged in the 130–360 ms region. The analyses on the values in the 360–900 ms region showed a main effect of switch type (*F*(1, 15) = 4.7, *p* = .047). This demonstrated the statistical reliability of the positive-going deflection for source switches.

To test whether these differences were due to mixing cost effects, rather than effects of the task and source switch themselves, the stay trials of source and task switching blocks were compared in the same intervals. No main effect of condition (*p* > .25) or electrode by condition interaction was found (*p *> .66), suggesting that sustained block differences are not driving the differences observed between source and task switches.

**Fig. 5:**
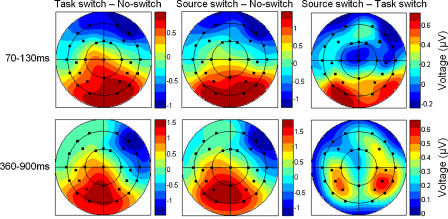
Scalp distributions of the ERP effects for task and source switches. Voltage spline maps illustrating the differences between task and no switches (left), source and no switches (middle), and source and task switches (right) in the 70–130 and 360–900 ms intervals. The maps are scaled to the minimum and maximum in each condition.

To assess whether the observed differences were qualitative or quantitative in nature, a final analysis focused on the scalp distributions of task and source switching effects. Fig. 5 shows the voltage spline maps of the ERP differences between task and no switches, source and no switches, and source and task switches in the 70–130 and 360–900 ms intervals. As can be seen, the scalp distributions in the early period appear virtually identical. The later distributions also seem similar, although the direct comparison between source and task switches suggests a somewhat more bilateral posterior focus for source switches. Statistically, none of the scalp distributions differed significantly. ANOVAs on data scaled to equate overall amplitudes across conditions (McCarthy and Wood, 1985) did not reveal significant interactions between switch type and electrode site for either the 70–130 or 360–900 ms intervals. This was true both when comparing the difference between task and no switches with that between source and no switches, and when comparing the former with the difference between source and task switches. To ensure that the lack of significant effects was not a result of statistical insensitivity due to entering all electrode sites into the analyses, we also compared task and source switches across the four frontal and four posterior electrodes where the differences were largest. Even with this liberal analysis, no statistically significant effects emerged (*p* > .118). Thus, although there were quantitative differences in the neural activity associated with source and task switching, this activity did not differ in kind.  

#### 3.2.3 Double switches

ERPs to double switches strongly resembled the ERPs elicited by task and source switches performed in isolation (see Fig. 3). Indeed, none of the statistical comparisons between double and either task or source switches were significant, with only the difference between double and task switches in the 360–900 ms interval approaching statistical significance (*F*(1, 15) = 4.4, *p* = .053).  

## 4 Discussion

The primary aim of the experiment was to determine whether the ability to configure appropriate task sets and the ability to select self-generated or perceptually derived information relevant to the current goals have similar, or distinct, neural bases. In this context, we used the term source switching to refer to the allocation of processing resources to perceptually derived (stimulus-oriented) or self-generated (stimulus-independent) information. The term task switching was used to refer to processes that may orient attention towards task-relevant properties of a representation built on the basis of perceptually derived or self-generated information, and to the choice of a response on the basis of the selected information. Behaviourally, switch costs were observed for all types of switches used in the experiment. Importantly, these costs did not differ between source and task switches, although participants were slower to perform the double switches. Relative to performing the same task on the same type of information, any type of switching was associated with distinct sets of neural activity at various points in time. Crucially, however, although these activities were engaged to different degrees, no qualitative differences were observed across switch types. Thus, the data suggest that task and source switching share a neural basis. We will first briefly discuss the ERP effects associated with switch trials, and then turn to the comparison between task and source switching.

Switch trials were associated with three distinct ERP effects, which reflected both switching processes and processes associated to the mixed task blocks. These effects were defined on the basis of task versus no switches, but were also present for source and double switches. The first effect consisted of a bipolar modulation over frontal and occipital scalp sites shortly after item onset. Although there are no standard item-related ERP task switching effects with which this finding can be compared, two previous studies of task switching have observed ERP modulations over posterior scalp sites shortly after the onset of the item initiating the switch (Karayanidis et al., 2003; Rushworth et al., 2005). Early visual ERPs over the back of the head can reflect the amount of attention paid to stimulus events (e.g. Natale et al., 2006; see Luck et al., 2000 for review). A possible interpretation of the early modulation observed here is that it reflects changes in the amount of attention paid to the information on the screen. Switch and stay trials alternated on a regular basis, and subjects thus knew what type of trial would come up next. This may have enabled an early recruitment of cognitive control processes to support all types of switching, at the expense of a reduction in the amount of attention paid to the external event. A recent ERP study of a stimulus–response conflict task also found that cognitive control mechanisms can affect very early stages of processing (Scerif et al., 2006). In that study, ERP modulations with bipolar distributions over frontal and occipital electrodes were observed at around 110 ms after stimulus onset.

The functional interpretation of the later two ERP effects is more difficult. In addition to the early effect, switch trials were associated with a centrally-distributed positive-going modulation around 300 ms, and a later sustained bipolar modulation over right frontal and occipital sites until at least 900 ms. Positive modulations just before 300 ms have been linked to a number of cognitive processes, including visual discrimination (Lindholm and Koriath, 1985), working memory (Gevins et al.*,* 1996; Smith, 1993; Van Petten et al., 1991) and emotion (Kissler et al., 2006). Higher cognitive processes have also been associated with late sustained ERP modulations (e.g. Friederici, 2004; Ruchkin et al., 2003; Rugg and Wilding, 2000). Importantly, the late effect is not simply a continuation of that seen shortly after item onset; the scalp distributions of the two effects differed. One possibility is that the late effect reflects the engagement of cognitive control processes. Although it is not possible to directly infer the intracerebral origins of scalp-recorded EEG, it is interesting to note that cognitive control relies on the frontal cortex (Ridderinkhof et al., 2004). The sustained negative modulation over frontal scalp sites also fits in well with previous ERP investigations of task switching. Despite the inconsistencies across studies, ERP modulations with a frontal focus in response to switch events have been found on several previous occasions (Lorist et al., 2000; Moulden et al., 1998; Poulsen et al., 2005; Rushworth et al., 2002; Wylie et al., 2003). In addition, frontal effects are regularly observed in the interval *preceding* task switches, in paradigms using pre-stimulus cues (Gladwin et al., 2006; Lorist et al., 2000; Goffaux et al., 2006, 2008; Travers and West, 2008). These effects may reflect changes in the anticipation of, and preparation for, an upcoming event (see Brunia and van Boxtel, 2001, for review). Although we did not use pre-stimulus cues in the present experiment, switch and stay trials alternated. The task set and orientation of attention established during a switch trial could therefore be re-used for the next (stay) trial. Actively maintaining these in preparation for the upcoming event was therefore beneficial.

Although we can only speculate about the precise functional role of the observed ERP differences related to switching, the important question we wished to address was whether there is any evidence from scalp-recorded ERPs that source and task switching are associated with similar, or distinct, neural activity. Previous fMRI studies have shown a consistent network of brain regions recruited during a range of task switching paradigms and one source switching experiment, although these two aspects of cognitive control have not previously been combined in a single experiment. Studies that have investigated task switching in isolation have consistently demonstrated a role of dorsolateral prefrontal cortex (DLPFC) and parietal cortex (DiGirolamo et al., 2001; Dove et al., 2000; Dreher et al., 2002; Kimberg et al., 2000; Ruge et al., 2005; Sohn et al., 2000; Wager et al., 2005). In the only existing fMRI study of source switching to date, Gilbert et al. (2005) observed that switching between perceptually derived and self-generated information also gives rise to changes in activity in DLPFC and parietal cortex. More generally, animal and human neuropsychological studies suggest that the prefrontal cortex is involved in representing task set or goal-directed information, and the superior parietal cortex in switching attentional focus (Miller and Cohen, 2001; Posner and Petersen, 1990). Thus, brain imaging evidence suggests that the DLPFC and parietal cortex may play a role in *both* task and source switching. The current results support the general notion that task and source switching share a neural basis by demonstrating a common electrophysiological signature for these two aspects of cognitive control in the first second following stimulus onset.

The role of another region of the prefrontal cortex in task and source switching, lateral rostral PFC, is less clear. Some fMRI task switching studies have found differential activity in this region (Braver et al., 2003; DiGirolamo et al., 2001; Dreher et al., 2002; Rushworth et al., 2002), but the majority has not (e.g. Brass and von Cramon, 2004; Klingberg et al., 2002; Ruge et al., 2005; Sohn et al., 2000; Pollmann, 2001). Importantly, effects in lateral rostral PFC were found for source switches by Gilbert et al. (2005). In combination with the presumed role of this region in self-generated thoughts (Burgess et al., 2005; Christoff and Gabrieli, 2000), Gilbert et al. therefore suggested that lateral rostral PFC supports the switching between stimulus-oriented and self-generated thoughts rather than the switching between task sets. Findings of lateral RPFC activations associated with task switching in other studies might have been related to the precise experimental designs, which may have led to more or less attending towards self-generated thoughts during task switches.

If lateral rostral PFC supports source, but not task, switching, it might be expected that qualitatively different patterns of electrical brain activity would be observed for each. In contrast, the present findings identified quantitative, but not qualitative, differences across these two aspects of cognitive control. None of the scalp distributions differed reliably between task and source switches. These null effects may be due to a general lack of statistical power in the current study. However, when the analyses were repeated on the subset of electrodes that showed the strongest effects to boost statistical sensitivity, the scalp distributions still did not differ significantly between source and task switching. Although it is of course possible that the neural activity that may have differentiated the two conditions (such as that arising from lateral rostral PFC) does not manifest itself in scalp-recorded EEG, the data provide no evidence for the idea that source and task switching engage different neural mechanisms within the first 900 ms after stimulus onset. Rather, at least under the conditions used here, source and task switching appear to have the same neural bases. The lack of significant differences between source and task switching might also explain why double switches did not differ from either simple switch.

In addition to the type of neural activity, the data provide no evidence that the time at which switch-related activity occurred differed across task and source switches. We hypothesized that source switching would affect early ERP modulations, corresponding to the early allocation of attention to perceptually derived or self-generated information. Task switching, on the other hand, would affect later ERP modulations involved in stimulus–response mappings. The results do not support this idea, as the timing of the three ERP effects was the same across conditions. Notably, very early effects of task as well as source switching were observed. As discussed above, the study used a predictable trial sequence. This was essential to reduce reliance on external information in the face of generating information internally. The predictable trial sequence may have encouraged the recruitment of cognitive control processes in an anticipatory manner. Such top–down control is known to be able to influence early perceptual processes (cf. Driver and Frith, 2000; Friston, 2005).

The similarities in type and timing of electrical brain activity associated with task and source switches in the time window studied here suggest that a fundamental switching mechanism was recruited in both cases. This mechanism itself consists of multiple cognitive and neural processes, as each type of switch was associated with three distinct ERP effects. The similarities observed here across task and source switches are all the more remarkable given that the two types of switching differ in many respects, including performance, mixing costs (Ruge et al., 2006), the task instructions, and the necessity to both generate a letter and perform a switch on half of the source switching trials. The use of a predictable trial sequence may have increased the likelihood of obtaining common ERP effects for task and source switches. In fMRI work, it has been shown that tasks that rely on predictable as opposed to unexpected events involve rostral PFC to a lesser degree (Koechlin et al., 2000). In future work, it would be of interest to contrast task and source switching in other types of paradigms.

Although no qualitative differences were observed, activity associated with source and task switching did differ quantitatively. The magnitude of an ERP modulation is traditionally thought to reflect the degree to which cognitive processes are invoked (Handy, 2004). According to this argument, the processes associated with the early and late deflections were engaged to different extents for source than task switches. Thus, the fundamental processes associated with switching behaviour can be invoked more or less extensively depending on the type of task and type of information involved in the switching.

It is important to note that the present study focused on early and mid-latency ERP modulations occurring within the first 900 ms after stimulus onset. No qualitative differences were observed between the ERP responses to task, source, and double switches within this period of time. However, it is possible that differences between the switch types only emerge in a later time period, after 1 s. The design of the current study was optimised to look at early latencies. In future work, it will be important to assess whether task and source switching may engage different neural mechanisms at the time of response preparation and response execution.

As explained in Section 1, we use the term ‘source switching’ to refer to changes in the source of the currently attended information, either perceptual or self-generated (Burgess et al., 2005, 2007). Distinctions between other types of attention and task switches have been investigated in the literature. Rushworth et al. (2002, 2005) and Ravizza and Carter (2008) proposed a distinction between attentional and intentional set switching, and perceptual and rule switching, respectively. Distinct types of neural activity were reported to be associated with each switch type. Other groups have recently studied the redirecting of attention to different aspects of a sensory stimulus (e.g. Pollmann et al., 2006; Töllner et al., 2008). In these studies, changes in the dimension used to select targets in a visual search task (shape or motion versus colour singleton features) were contrasted with changes in response. Again, qualitatively different patterns of fMRI activity (Pollmann et al., 2006) and ERPs (Töllner et al., 2008) were observed for these two switch types. The above results contrast with the common ERP effects found for task and source switching in the current experiment. In our paradigm, the type of switch was independent from a repeat or change of the response key, and both tasks required participants to attend to the shape of a letter, whether it was presented on the screen or imagined. The differences between source and task switching have thus been minimized along these factors. When the focus is on higher-level cognitive control, and not on the comparison between visual properties or motor responses, electrophysiological switching effects do not appear to differ.

In conclusion, this study contrasted for the first time the neural correlates of two cognitive control functions that have thus far been studied independently. ERPs were used to investigate transient changes in neural activity while subjects switched between attending to perceptually derived and self-generated information, or switched between tasks involving different stimulus–response mappings. Robust ERP effects were found for all types of switching. However, these effects did not differ in terms of their time of occurrence or distribution over the scalp during the first 900 ms after stimulus onset. These results suggest that the two types of switching investigated here are represented, at least in part, by a common neural signature.
